# A Novel Patient-oriented Tool for Evaluating Quality Measurements

**DOI:** 10.7759/cureus.7726

**Published:** 2020-04-18

**Authors:** Sarah Rawi, Alec Freling, Adam Hemminger, Mark Wendling

**Affiliations:** 1 Internal Medicine, University of Connecticut Health Center, Hartford, USA; 2 Emergency Medicine, University of Connecticut Health Center, Hartford, USA; 3 Emergency Medicine, Virginia Commonwealth University, Richmond, USA; 4 Family Medicine, Lehigh Valley Health Network, Allentown, USA

**Keywords:** quality, patient-oriented, standardization, measurement

## Abstract

Quality measurements (QMs) have emerged as quantitative tools for measuring “quality”, an elusive term that has been historically difficult to define and quantify. However, current literature has demonstrated that these measurements are flawed. The purpose of this study was to identify the strengths and weaknesses of quality measurements and provide a novel scorecard for evaluating quality measurements. In this retrospective analysis, 246 quality measurements that are integrated into the most significant payer-provider contracts within our institution were analyzed. Each measurement was dissected based on type of measurement, evidence, precision, data exchange, alignment, and how patient-oriented. Our research showed a significant lack of quality measurement alignment across payer-provider contracts. As such, we developed and proposed a Quality Measurement Evaluation Tool (QMET) that scores a quality measurement’s ability to 1) reflect population health and 2) promote patient-oriented goals. Our research demonstrated the majority of quality measurements scored in the inadequate range (i.e., QMET score <6) and only few in the optimal range (i.e., QMET score 10-12). QMET provides a standardized and comprehensive method for appraising quality measurements, promoting continued use of QMs that accurately reflect population health and promote patient-oriented measurements. Future research into the application and reliability of QMET is needed.

## Introduction

For decades, health care organizations have struggled to measure “quality” - an elusive term that has been historically difficult to define and quantify. More recently, quality measurements (QMs) have emerged as quantitative tools for measuring quality and have been widely adopted by providers, hospitals, and health care systems across the country [1]. However, previous research demonstrates that these measurements are flawed, often struggling to adequately measure quality due to shortcomings in several key areas, such as data collection and clinical evidence [1-5]. Our study analyzes these key areas within our own organization and provides examples of such shortcomings. Furthermore, our study proposes a novel patient-oriented scorecard for reproducible analysis of quality measurements. The scale emphasizes the need for a movement towards patient-oriented quality measurements, a concept that is underrepresented and undervalued [5]. The ultimate goal of our study is to encourage adoption of patient-oriented quality measurements, which aim to understand and evaluate the definition of “quality” from the patient’s perspective.

## Materials and methods

This was a retrospective analysis of 246 quality measurements within a single metropolitan-area institution in Pennsylvania. Since 1993, our institution’s non-for-profit subsidiary includes 700+ physicians within 45 diverse medical specialties throughout 160 offices in Allentown and Bethlehem, Pennsylvania. There are over 50 payer-provider contracts within this institution, and quality measurements are integrated into these contracts [6]. To further understand the connection between quality metrics and patient-oriented care, this research analyzes the 10 largest contracts within this network. Each measurement within these contracts was dissected based on type of measurement, evidence, precision, data exchange, and alignment. More detailed definitions of these categories are shown in Table 1.

**Table 1:** Definitions Key

Term	Definition
Measurement Type	Whether a measurement is based on a series of actions that lead to a potential health benefit (process) or a measurement based on quantitative results (outcome).
Precision	A measurement with appropriate numerators/denominators, with proper exclusions and inclusions.
Data Exchange	Data shared from payer to provider or vice versa (unidirectional), or equal exchange of data between payer and provider (bidirectional).
Evidence	Measurement with clear evidence to support its validity (e.g., USPSTF Grade A or B; ACC/AHA high-moderate level evidence).
Alignment	Prevalence of measurement among payers (i.e., percentage of payers using given measurement).
Patient-Oriented	Measurement is of direct value to the patient (e.g., preventing vision loss or stroke).

## Results

Type of measurement

Our study categorizes the quality measurements into three types: surrogate outcomes, process, or mixed. Definitions and examples of each category are shown in Table 2. Of the 246 quality measurements, there are 192 process measures, 42 surrogate outcome measures and 12 mixed measures. This breakdown demonstrates the focus on measuring quality through process measures as opposed to surrogate outcomes or mixed measures. Using process or mixed outcomes does not adequately portray the health of this patient population. These measurements help form attainable goals for physician groups, but cannot accurately assess the health of their patients. Not only are majority of these quality measurements non-outcome based, the measurements that are outcome-based are only intermediate outcomes and are not patient-oriented. If a quality measurement was truly measuring what is important to the patient’s health, then it should not measure the quantitative values in their patient charts, but the symptomatic changes they live with. For example, it should not be measuring how many individuals have an HbA1c <9% or percentage of women who had a mammogram to screen for breast cancer within the past 24 months, but instead should be measuring how many of their diabetic patients have retinopathy and percentage of individuals with breast cancer with poor quality of life [7,8]. There are some measurements that are ‘mixed’, such as measuring individuals with diabetes who received a retinal or dilated eye exam or a negative exam in the year prior [9]. Only if these changes were made would we have patient-oriented quality measurements.

**Table 2:** Type of Measurement

Type of Measurement	Definition	Example
Surrogate Outcome	Quantitative results that have a measurable value related to a treatment modality or preventative care	Percentage of patients aged 18 to 75 years of age with diabetes mellitus who had HbA1c ⪯ 9% [7].
Process	Measurement of a series of actions that lead to a potential health benefit	Percentage of women aged 40 to 69 years who had a mammogram to screen for breast cancer within 24 months [8].
Mixed	The combination of both a process and a surrogate outcome	Percentage of members 18-75 years of age with diabetes (type 1 and 2) who received a retinal or dilated eye exam during the measurement year or a negative retinal or dilated eye exam in the year prior to the measurement year [8,9].

Evidence

The adoption of quality measurements is based upon clinical evidence and support from national criteria, such as the United States Preventive Service Task Force (USPSTF). However, our research indicates that while a large percentage of QMs are evidence-driven, a substantial portion of QMs is driven by payer data and/or expertise. For example, cervical cancer screening is strongly evidence-based as it measures the number of female patients aged 21 to 65 years who received cervical cytology in the past three years, a criteria endorsed by USPSTF and supported in clinical literature [10-11]. Conversely, quality measurements that monitor comprehensive control of diabetes do so by measuring the percentage of diabetic patients that received low-density lipoprotein (LDL) testing in the last year. There is limited clinical evidence to support the efficacy of these measurements, and national agencies do not support the claim that these measurements improve quality. As such, the failure of quality measurements to be both evidence-based and improve quality once again brings up the question of patients’ values. Patient-oriented quality measurements should have clinical evidence to support their efficacy in improving patient care and outcomes; otherwise, they are of no benefit to the patient.

Precision

QMs are quantified by a ratio: a numerator consisting of the number of patients appropriately meeting the measurement divided by a denominator of every patient that qualifies for the measurement. For QMs to reflect population health, it is essential that the way they are quantified is also precise. An example of a precise QM is prescribing controller medications for asthma. Asthma severity is clearly defined in medical practice, and providers prescribe controller medications according to severity - there is little variation in asthma management across providers or payers [12]. However, our research indicates that certain measurements are imprecise, such as cervical cancer screening. When measuring cervical cancer screening, multiple payers define the denominator as “women 24-64 years as of the last measurement year”; however, it does not exclude women who have a history of a hysterectomy, invalidating the denominator and reducing the measurement’s precision [12].

Furthermore, transfer and leakage are two other causes of poor precision. Transfer is the movement of patients between payers. Our research indicates that a patient with a prior hysterectomy, when transferred into this network, is inappropriately included in the denominator due to non-transferrable payer codes. The work-around for this problem is to manually input the appropriate code; however, this extra step is rarely carried out, leaving the patient in the denominator and skewing the data. Secondly, leakage causes further inaccuracies. For instance, the quality measurement on diabetic eye exam screenings has been especially difficult for this network. This measurement requires patients with diabetes from age 18 to 75 years old to receive an eye screening for retinal disease [9]. Unfortunately, patients often travel out-of-network and are missed by our network, resulting in improper reimbursements and inaccurate population data [13].

Our research demonstrates the shortcomings in properly defining quality measurements. This challenges the success of quality measurements in reflecting quality and population health. The lack of a standard procedure for creating quality measurements - or a method for revising their flaws - results in an inability of providers to tailor medical services. This inability hinders the movement toward the goal of patient-oriented quality measurements.

Data exchange

Our research also pinpoints several other areas in which quality measurements struggle to accurately measure the quality of health care systems. For example, electronic medical record (EMR) systems - in their current form - have barriers to inputting data and properly documenting patient information. For example, this institution’s EMR system can only query data for diabetic foot exams if it is placed in a particular field and will not be queried if it is written in narrative form, which deflates the rate of diabetic foot exams.

Furthermore, this network utilized three types of data extraction: insurance claims, clinical reports, and self-reporting. These different forms of data distribution result in the unidirectional (i.e., from payer to provider or vice versa) or bidirectional (i.e., equal exchange of data between payer and provider) sharing of data. For example, the QM measuring body mass index (BMI) is bidirectional as the provider shares its clinical data with the payer and the payer shares its claims data with the provider. The QM measuring diabetic foot exams is unidirectional as only the provider shares clinical data collected on foot exams to the payer. In the future, every provider-payer relationship should adapt to shift the culture from unidirectional data exchange to bidirectional data exchange in order to best assess population health. Without this shift, the healthcare system will continue to fail to reflect population health simply due to a lack of communication between providers and payers.

Alignment

Arguably the greatest issue facing quality measurements is the lack of alignment. This research finds significant variation in type of measurement, evidence, precision, and data exchange. Of the 246 QMs analyzed in this study, there were only three quality measurements that were utilized by more than 75% of payers. ‘Breast Cancer Screening’ is an example of a quality measurement with strong alignment as it was standardized across 77% of the payers within this study. On the other hand, ‘Fall Risk Assessment for Older Adults’ is a quality measurement that is included in only 31% of contracts. This misalignment of quality measurements across payers is unacceptable when considering the goal of patient-oriented quality care. Significant improvement is needed in order to provide patients with quality care that is uniform across payers, such that patients can maneuver between insurance plans without concern for drastic changes in quality.

Limitations

This analysis was limited by the nature that it was retrospective. The analysis was also limited to one health network, and thus might not apply to all networks in various locations. The analysis was also only completed on select payers within the network, therefore, may not apply to all payers.

## Discussion

Our solution to the issue of alignment is a novel scoring system for objectively grading quality measurements. Quality Measurement Evaluation Tool (QMET), shown in Table 3, is a 12-point scoring system that evaluates quality measurements based on the areas described in our study. Further examples of each category are listed in Table 4. The scoring criteria is as such that less than six points is an inadequate measurement (i.e., should be revised or reconsidered), six to seven points is a mediocre measurement (i.e., should be re-evaluated for efficacy), eight to nine points is an adequate measurement (i.e., should be used for internal quality improvement), and ten to twelve points is an optimal measurement (i.e., should be used for universal quality improvement).

**Table 3:** Quality Measurement Evaluation Tool (QMET)

Factor	Score			Subtotal
Measurement Type	0 - Process measurement	1 - Mixed measurement	2 - Outcome measurement	__/2
Precision	0 - Not precise	1 - Precise but exclusions need further definition	2 - Precisely defined with proper exclusion	__/2
Evidence	0 - No evidence or minimal evidence	1 - Contradicting or controversial evidence	2 - Clear and consistent evidence	__/2
Data Exchange	0 - Unidirectional (payer to provider OR provider to payer)		2 - Bidirectional (payer to provider AND provider to payer)	__/2
Alignment	0 - QM is used in less than 50% of insurance contracts	1 - QM is used in 50-74% of insurance contracts	2 - QM is used in at least 75% of insurance contracts	__/2
Patient-Oriented	0 - Not patient-oriented	1 - Limited or questionable value for patient	2 - Patient-oriented	__/2
			Total:	__/12

**Table 4:** Example Evaluations

Quality Measurement	Measurement Type Score	Precision Score	Evidence Score	Data Exchange Score	Alignment Score	Patient-Oriented Score	Total Score	Qualitative Analysis
Diabetic Foot Exams	0	2	1	0	0	0	3/12	Inadequate
Colorectal Cancer Screening	0	2	1	2	2	1	8/12	Adequate
Hospital Consumer Assessment of Healthcare Providers and Systems (HCAHPS)	2	2	2	2	2	2	12/12	Optimal

Colorectal cancer screening scores 8/12 due to strong performances in the areas of alignment, precision, evidence, and how patient-oriented. HCAHPS receives a perfect score and serves as an example of an optimal quality measurement; however, our study finds few quality measurements that meet these criteria, demonstrating the significant need for further development.

QMET focuses on two important elements of quality measurements: population health and patient-oriented goals. The first four factors listed in the scale - measurement type, precision, evidence, and data exchange - emphasize the importance of utilizing quality measurements that fulfill the requirement necessary to reflect population health. Fulfilling these requirements allows quality measurements to accurately measure and analyze population health. It also provides healthcare organizations with data to help drive organizational decisions that will best improve the health of their patients. QMET helps achieve the original goal of quality measurements, which is to advance the quality of our healthcare system. Patient-oriented quality measurements position the patient at the center of how we define quality. They are respectful and responsive to the individual needs, values, and preferences of each patient. The success of each measurement is linked to how well it meets the patient’s own definition of quality [14]. This is a dramatic shift away from our previous understanding of quality, which focused predominantly on population health and provider performance.

## Conclusions

Our research analyzes the failure of quality measurements to accurately represent population health and promote patient-oriented goals. These failures are derived from shortcomings in the following domains: measurement type, precision, evidence, data exchange, and alignment. We propose Quality Measurement Evaluation Tool (QMET) as a method for evaluating and critiquing quality measurements. QMET provides a reproducible and objective tool for choosing which quality measurements need reconsideration, revision, or are ready for implementation.
